# The effects of interventions to enhance cognitive and physical functions in older people with cognitive frailty: a systematic review and meta-analysis

**DOI:** 10.1186/s11556-022-00299-9

**Published:** 2022-08-24

**Authors:** Ada Chung Ying Tam, Amanda Wan Yee Chan, Daphne Sze Ki Cheung, Lily Yuen Wah Ho, Angel Shuk Kwan Tang, Martin Christensen, Mimi Mun Yee Tse, Rick Yiu Cho Kwan

**Affiliations:** 1 Queen Elizabeth Hospital, Hospital Authority, Hong Kong, China; 2 School of Nursing and Health Studies, Hong Kong Metropolitan University, Hong Kong, China; 3 Centre for Gerontological Nursing, School of Nursing, The Hong Kong Poltechnic University, Hong Kong, China; 4 School of Nursing, The Hong Kong Polytechnic University Centre for Gerontological Nursing, The Hong Kong Polytechnic University Hong Kong, Kong, China; 5 School of Nursing, Caritas Medical Centre, Hong Kong, China; 6 School of Nursing, Tung Wah College, Ma Kam Chan Memorial Building,31 Wylie Road, Homantin, Hong Kong, China

**Keywords:** Cognitive frailty, Intervention, Review, Older adults

## Abstract

**Introduction:**

Cognitive frailty is the co-existence of mild cognitive impairment and physical frailty that increases the risk of adverse health outcomes. The existing systematic reviews on cognitive frailty in the literature have focused only on identifying associated factors and adverse outcomes, and their relationship with frailty and cognition. This study aimed to examine the effects of interventions on cognitive functions, frailty, and physical functions and provide an overview of intervention components used in older people with cognitive frailty.

**Methods:**

This is a systematic review and meta-analysis. Medline, PubMed, CINAHL, Embase, PsycINFO, and Cochrane were searched for publishing during 2013–2021. Studies were selected based on the following eligibility criteria: 1) older people (age ≥ 60 years), 2) cognitive frailty, 3) outcomes on frailty or cognition or physical function, and 4) randomized controlled trial with any type of intervention. The Physiotherapy Evidence Database (PEDro) scale was used to rate the quality of the included studies. The review protocol was registered with PROSPERO (CRD42021251321).

**Results:**

Two thousand five hundred six studies were identified, 9 were eligible, and 8 were included in the meta-analysis. The standardized mean difference (Hedges G) between groups of cognitive functions was 0.95, frailty status was 0, physical function in walking was -1.67, and the physical function in core strength assessment was 3.39. Physical activity appeared as an essential component in all interventions for older people with cognitive frailty.

**Discussion:**

All interventions include physical activity as one of the components. Other components include cognitive training, nutrition education, behavioural intervention, mind–body intervention, psychosocial support, and virtual reality. The interventions are effective to promote cognitive and physical functions, but not physical frailty.

**Supplementary Information:**

The online version contains supplementary material available at 10.1186/s11556-022-00299-9.

## Introduction

Cognitive frailty is the coexistence of physical frailty and mild cognitive impairment (MCI) such that the cognitive impairment is not severe enough to meet the diagnostic criteria for dementia [1]. Physical frailty is an intermediate state between normal functioning and disability [2, 3]. It is also a phenotype characterized by weight loss, fatigue, exhaustion, weakness, low physical activity, slowness, and mobility impairment [4]. Cognitive frailty is prevalent in community-dwelling older people, with prevalence rates ranging from 4.4% to 19.9% [5, 6]. Physical frailty and cognitive impairment are interrelated, as they share similar precipitating factors and pathogenesis pathways, such as sarcopenia and physical inactivity [7, 8]. Compared with physical frailty alone or mild cognitive impairment alone, cognitive frailty is associated with a higher risk of many adverse health outcomes, such as dementia, poor quality of life, fall risk, mortality, hospitalisation, and dependency [6, 9–14].

Unlike dementia, cognitive frailty is potentially reversible [11, 14]. It is the result of a decrease in cognitive reserve, which is not part of normal ageing [1, 10]. Factors associated with cognitive frailty could be classified as modifiable or non-modifiable [3, 14]. Socioeconomic status such as level of education and income, are non-modifiable associated factors of cognitive frailty [15]. In contrast, physical inactivity (e.g., lack of exercise), malnutrition, lack of cognitive stimulation, psychological factors (e.g., self-esteem) [16], medication [17] and social contact [1, 8, 10, 18] are modifiable associated factors. Modifying these factors may ameliorate the progression of cognitive frailty [19] and reduce its adverse outcomes [10].

The preliminary evidence has shown that physical activity, changes in behaviour, health and social care provision, cognitive training, and nutrition interventions produced positive effects on cognitive frailty [20–25]; however, the effects between studies have been inconclusive. Several dietary components and patterns [26] and physical function [27] were found to have a strong association with cognitive frailty. Exercise and nutrition may improve cognitive functions, physical functions, and frailty status for frail older people [28–30]. Intervention components in different studies varied; however, the intervention components employed to promote the health of older people with cognitive frailty have not been systematically examined.

The existing systematic reviews on cognitive frailty in the literature have focused only on identifying associated factors and adverse outcomes [6, 9, 31], and their relationship with frailty and cognition [32–34]. There are no systematic reviews evaluating the effects of the interventions on older people with cognitive frailty. Cognitive frailty is a major health issue for older people. It is essential to identify effective intervention components to design future interventions to treat cognitive frailty. Therefore, the aims of this review were to:

1. Provide an overview of intervention components used in older people with cognitive frailty, and
2. Examine the effects of interventions on cognitive functions, frailty, and physical functions in older people with cognitive frailty

## Methods

This is a systematic review and meta-analysis. The Preferred Reporting Items for Systematic Reviews and Meta-Analyses (PRISMA) [35] was used as the format to guide and report the outcomes of this review. The review protocol was registered with PROSPERO (CRD42021251321).

### Eligibility criteria

Studies were selected based on the following eligibility criteria: (1) older people (i.e., enrolled participants with a mean age of ≥ 60 years), (2) with cognitive frailty, (3) had any outcomes on frailty or cognition or physical function, and (4) the use of a randomized controlled trial with any type of interventions.

### Information sources

Six electronic databases (CINAHL, Cochrane, Embase, PsycINFO, Pubmed, and Medline) were searched for relevant studies from 1^st^ January 2013 to 11 September 2021. We limited the search started from 2013 onwards because this is the first mention of the concept of cognitive frailty by the International Academy on Nutrition & Ageing (IANA) / International Association of Gerontology and Geriatrics (IAGG) International Consensus group (1).

### Search strategy

The search was based on the following three groups of keywords: (1) “cognitive frailty” or “cognitive impairment”, (2) “frail”, and (3) “older people”. The search strategies used in each of the specific databases are presented in Additional file 1. In addition, manual searches of the reference lists of relevant articles were conducted and all eligible studies were searched to identify other trials. We did not specify the types of interventions and outcomes measured in the literature search to ensure that all interventions for cognitive frailty were included.

### Selection process

Identified articles were imported into Clarivate Analytics Endnote X9.0. Duplicates were removed by Endnote. Two researchers independently screened the articles against the inclusion criteria in two steps: titles and abstracts, followed by full texts. In cases of disagreement, two researchers discussed until a consensus was reached. In cases where disagreement could not be solved, a third researcher would be consulted.

### Data collection process

Data were copied to a pre-designed data extraction form using Microsoft Excel. If there were disagreements over the extracted data, the third researcher was invited for discussion. In case of queries, the authors would be contacted.

### Data items and effect measures

To obtain a profile of the studies, the following information was extracted: authors, year of publication, age of the participants, sample sizes, population characteristics, intervention strategies, controlled conditions, outcomes, and data collection time points.

To examine the effect of an intervention on frailty, physical and cognitive functions, values quantifying frailty, physical and cognitive functions were extracted, such as frailty score, cognitive examination, muscle strength, physical activity, and physical function. Also extracted were values of the outcome variable (i.e., mean, standard deviation, and sample size in each group) at baseline (T0) and at the time point after the completion of the intervention (T1) in both the intervention and control groups.

### Assessment of the risk of bias in the included studies

The Physiotherapy Evidence Database (PEDro) scale [36] was used to rate the quality of the included studies. The PEDro scale is comprised of 11 dichotomous items: eligibility criteria, randomization, concealment, baseline, blinding of subjects, therapists and assessors, subjects retention, intention to treat analysis, between-group comparison, and measures of variability. The item for eligibility criteria was not scored, therefore, for the remaining items one point for all ten items added up to a total score. The quality of the RCT was rated as excellent (PEDro = 9 – 10), good (PEDro = 6 – 8), fair (PEDro = 4 – 5), or poor (PEDro < 4). To ensure at least fair methodological quality, only studies with a PEDro score of ≥ 4 were included in the quantitative synthesis (i.e., a meta-analysis of the effects) [36].

### Synthesis methods

To summarize, details of the intervention, such as type, materials used, providers and mode of delivery, and intervention outcomes were explored.

The Cochrane Handbook for Systematic Reviews was used to guide the handling and analysis of the data [37]. Both between-group and within-group effects were summarized using Hedges G (taking 0.2, 0.5, and 0.8 as the respective thresholds for small, medium, and large effects) and a 95% confidence interval.

To evaluate the between-group effects, a meta-analysis was performed if three or more studies measured the same type of outcome, and if the mean and standard deviation of the outcome at T1 were provided. The results of the meta-analysis are presented through Forest plots using RevMan version 5.3. The heterogeneity of the studies was indicated by the I^2^ index, taking 75%, 50%, and 25% as the respective thresholds for high, medium, and low ratios of interstudy heterogeneity [37]. Random effect models were used because the components of the intervention were not identical [38].

## Results

### Study selection

**Fig. 1:**
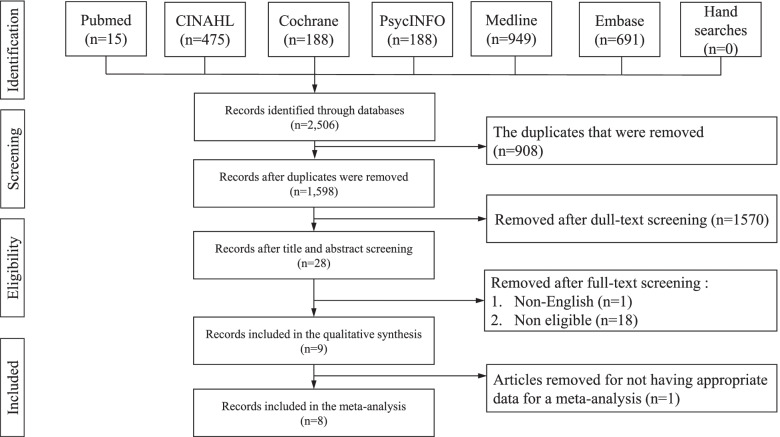
PRISMA flowchart

Two thousand five hundred six articles were identified in the selected databases: Pubmed (*n* = 15), CINAHL (*n* = 475), Cochrane (*n* = 188), PsycINFO (*n* = 188), Medline (*n* = 949), and Embase (*n* = 691) (Fig. 1). Nine hundred and eight duplicated articles were removed. After screening the titles and abstracts, a further 1,570 articles were removed. Nineteen articles were found to be ineligible and were removed after the full-text screening. Nine articles were eligible for qualitative synthesis. Only eight articles [20, 21, 23–25, 39–41] were included in a meta-analysis of different outcomes because a study did not provide the mean and standard deviation of the outcomes and was excluded [22].

### Study characteristics

Nine eligible articles were RCTs that evaluated the effects of interventions on a population of 1,707 older people with cognitive frailty (Table 1). The mean age of the participants ranged from 67.7 to 79.1 years. In seven studies no special health condition was targeted. Two studies targeted inactive older people. Eight studies (*n* = 8, 88.9%) included older people who were pre-frail or frail, and one study (*n* = 1, 11.1.%) recruited frail older people only.

**Table 1:** Summary of studies about interventions for managing cognitive frailty in older people

**No**				**1st Author**	**Year**	**Sample Size**	**Age (year)** **Mean/Median** ** ± SD/(IQR)**	**Population**	**Name of Intervention**	**Control**	**Outcome**	**Time**^**c**^
1				Liu	2018	*N* = 1,298 I:644 C:654	I:78.9 ± 5.3 C:79.1 ± 5.2	Age = 70–79 Healthy Sedentary Pre-frail/frail and MCI	LIFE	AC (Health education)	IM (IL6)	T1:24 months
2				Yoon	2018	*N* = 65 I: 32 C: 33	I:73.82 ± 4.37 C:74.03 ± 4.27	Age ≥ 65 Healthy Pre-frail/frail and MCI	Resistance exercise training	AC (balance and resistance band stretching)	Co (MMSE, FAB, CERAD, TMT, DST, RM) F (FFI) PF (TUG, SPPB, GS; HG, PT)	T1:16w
3				Furtado^a^	2020	*N* = 60 I:20 C(CSE):21 C:19	I:80.14 ± 8.19 C(CSE):81 ± 4.79 C:80.93 ± 10.01	Age ≥ 75 Pre-frail/frail and MCI Women	Chair multimodal exercise	UC AC (CSE)	IM (IL6) PF (SFTB) N (MNA)	T1:28w
4				Kwan	2020	*N* = 33 I:16 C: 17	I:70.5(7) C:71.0(14)	Age ≥ 60 Healthy Pre-frail/frail and MCI MVPA: < 150 min in the last 7 days	mHealth brisk walking intervention	AC (health education, brisk walking training, conventional behavioural intervention)	Co (MoCA) F (FFI) PA (WT, SC, MVPA) PF (PASE, HG, GS)	T1:13w
5				Lee	2020	*N* = 42 I: 18 C:22	I:73.7 ± 4.6 C:74.2 ± 4.4	Age ≥ 65 Healthy Frailty and MCI	High-speed power training	AC (health education)	Co (MMSE, FAB, FLT) F (FFI) PF (TUG, GS, PT, and RTD)	T1:8w
6				Chen	2021	*N* = 62 I:31 C:31	I:84.6 ± 4.2 C:84.8 ± 5.4	Age > = 75 Healthy Pre-frail/frail and MCI	Otago exercise programme	AC (health education)	PF (FTSST, TUG, BBS) PS (GDS-15, SF-12 MCS)	T1:12w
7				Jiayuan^b^	2021	*N* = 93 I:31 C(TCC): 31 C(M):31	I (MTCC):71.3 ± 5.0 C(TCC):71.7 ± 3.9 C:70.8 ± 4.2	Age ≥ 65 Healthy Pre-frail/frail and MCI	Tai Chi Chuan, mindfulness intervention	AC (M or TCC)	Co (MMSE) PF (SPPB, TUG, 30 s-Chair Test)	T1:12 m
8				Murukesu	2021	*N* = 42 I:21 C:21	I:67.7 ± 4.4 C:70.8 ± 7.1	Age ≥ 60 Healthy Pre-frail/frail and MCI	Multi-domain intervention	UC	PA (IPAQ) PF (FAQ) PS (FS, GHQ-12, COPE)	T1:24w
9				Kwan	2021	*N* = 17 I:9 C:8	I:73(7.5) C:77.5(15.3)	Age ≥ 60 Healthy Pre-frail/frail and MCI Outdoor walker	VR reality motor-cognitive training	AC (PA and cognitive training)	Co (MoCA) F (FFI) PF (TUG, HS)	T1:8w

*AC* Active care, *C* Control group, *CERAD* Consortium to Establish a Registry of Alzheimer’s Disease, *CMC* Chair Multimodal Exercise, *Co* Cognition, *COPE* Coping Orientation to Problems Experienced, *CSE* Chair Elastic-band Muscle Strength Exercise, *DS* Digit span test, *F* Frailty status, *FAB* Frontal Assessment Battery, *FAQ* Functional Activity Questionnaire, *FFI* Fried Frailty Index, *FLT* Frontal lobe test, *FTSST* Five times sit to stand test, *GDS-15* Geriatric Depression Scale, *GHQ-12* General health questionnaire, *GS* Gait speed, *HG* Hand grip strength, *IM* Immune marker, *I* Intervention group, *IPAQ* International physical activity questionnaire, *LIFE* Lifestyle interventions and independence for elders, *M* Mindfulness, *MTCC* Mindfulness-based Tai Chi Chuan, *MMSE* Mini Mental State Examination, *MNA* Mini Nutritional Assessment, *MoCA* Montreal Cognitive Assessment, *MVPA* Moderate to vigorous physical activity, *N* Nutrition, *PA* Physical activity, *PASE* Physical Activity Scale for the Elderly, *PF* Physical function, *PT* Peak torque, *RM* Rey 15-item memory test, *RTD* Rate of torque, *SF-12 MCS* 12-item Short Form Health Survey Mental Component Summary, *SFTB* Senior Fitness Test Battery, *SPPB* Short Physical Performance Battery, *SC* Step count, *TCC* Tai Chi Chuan, *TMT* Trail Making Test, *TUG* Time Up and Go Test, *UC* Usual care, *VR* Virtual reality, *WS* Walking time

^a^ CME used as the main intervention group

^b^ Mindfulness intervention used as the control group and MTCC as the main intervention group

^c^ T1 refers to the time point after the completion of the intervention

The duration of the interventions ranged from 8 weeks to 24 months. Five studies (*n* = 5, 55.6%) reported a single-domain intervention and four studies (*n* = 4, 44.4%) involved an intervention with two or more domains conducted simultaneously. For example, one study reported using both an mHealth behavioural change approach and brisk walking in the intervention group [20], while a multi-domain intervention reported in another study included physical activity, cognitive training, dietary counselling, and psychosocial support [23].

All studies reported included a component of physical activity. Types of exercise included high-speed power training, high-speed resistance training, balance, flexibility and strength training, brisk walking, Tai Chi Chuan, cycling, and Otago exercise. One study included a mindfulness element in the Tai Chi Chuan intervention [40]. One study involved physical and cognitive intervention components simultaneously in a virtual reality (VR) platform [41]. Nearly half of the studies used health education and counselling as the active control (*n* = 4, 44.4%). One study (*n* = 1, 11.1%) used balance and resistance band stretching as the active control, and one used physical and cognitive training simultaneously without a VR platform as the active control (*n* = 1, 11.1%). Two studies used the usual care group as the control (*n* = 2, 22.2%), and one study (*n* = 1, 11.1%) used mindfulness as the control. All studies reported the immediate post-intervention effects. However, two studies had examined the effects at a mid-point during the intervention period in order to track the trajectory of change (*n* = 2, 22.2%) [24, 39].

The majority of the studies (*n* = 5, 55.6%) measured cognitive functions using the Mini-Mental State Examination (MMSE), Frontal Assessment Battery (FAB), Trail Making Test (TMT), Digit Span Test, and Montreal Cognitive Assessment (MoCA) [20, 21, 24, 40, 41]. Three studies (37.5%) examined frailty status using the Fried Frailty Index (FFI) [24, 25, 41].

Eight studies included in quantitative synthesis had measured physical functions (*n* = 8, 100%), using different tools, including the Time Up and Go Test (TUG), handgrip strength, gait speed, peak torque, rate of torque, the Functional Activity Questionnaire (FAQ), the Senior Fitness Test Battery (SFTB), the Physical Activity Scale for the Elderly (PASE), and the Short Physical Performance Battery (SPPB). Two studies (*n* = 2, 25.0%) evaluated physical activity in terms of time spent on walking, step count, and moderate-to-vigorous physical activity (MVPA) by using accelerometers and the International Physical Activity Questionnaire (IPAQ).

### Risk of bias in the studies

The PEDro total scores of the eight articles ranged from 5 to 8 (Table 2). One article (12.5%) was rated as fair and seven articles (87.5%) were rated as being of good quality.

**Table 2:** Risk of bias in individual studies as measured using the PEDro scale

No	Authors	Year	Eligibility	Random allocation	Concealed	Baseline similarity	Blinding(P)	Blinding (T)	Blinding (A)	Dropout	ITT	Group comparison	Point measures and variability data	PEDro total score	Quality rating
1	Yoon	2018	Y	Y	N	Y	N	N	N	N	Y	Y	Y	5/10	Fair
2	Furtado	2020	Y	Y	Y	Y	N	N	Y	Y	Y	Y	Y	8/10	Good
3	Kwan	2020	Y	Y	Y	Y	N	N	Y	Y	Y	Y	Y	8/10	Good
4	Lee	2020	Y	Y	Y	Y	N	N	N	Y	Y	Y	Y	7/10	Good
5	Chen	2021	Y	Y	Y	Y	N	N	Y	Y	N	Y	Y	7/10	Good
6	Jiayuan	2021	Y	Y	Y	Y	N	N	Y	Y	N	Y	Y	8/10	Good
7	Murukesu	2021	Y	Y	Y	Y	N	N	Y	Y	Y	Y	Y	8/10	Good
8	Kwan	2021	Y	Y	Y	Y	N	N	Y	Y	Y	Y	Y	8/10	Good

*Y* Yes, *N* No, Score ≤ 3 poor, 4-5 fair, 6-10 good

### Objective one: provide an overview of intervention components used in older people with cognitive frailty

As shown in Table 3, the intervention components of the included studies were categorized by 1) type of intervention, 2) materials used, 3) provider, 4) mode of delivery, 5) tailoring, and 6) dosage.

**Table 3:** Summary of the interventions

Study Profile			Intervention Components					
**No**	**Author**	**Year**	**Type**	**Material used**	**Provider**	**Mode of delivery**	**Tailoring**	**Dosage (C: course; F: frequency (per wk); D: duration)**
1	Liu et al	2018	PA (multi-component exercise^d^) Health education	Chair Weight-bearing device	Not mentioned	Individual training	Physical fitness (RPE^e^)	C:24 months F:5–6 (2 centre visits & 3–4 home based visits) D:55 min
2	Yoon	2018	PA (strengthening exercise)	Elastic band	Exercise specialists	Individual training	Physical fitness (RPE^e^)	C:16 wks F:3 D:Not specified
3	Furtado	2020	PA (strengthening exercise^a^)	Chair Elastic band	Exercise specialists	Group class	Physical fitness, (HRmax)	C:28 wks F:2–3 D:Not specified
4	Lee	2020	PA (high-speed power exercise)	Elastic band	Exercise specialists	Group class	Physical fitness (RPE^e^)	C:8 wks F:3 D:Not specified
5	Kwan	2021	PA (brisk walking) Behavioural intervention	Technology device	Non specialist	Individual training	Physical fitness (baseline level of fitness)	C:12 wks F:3–10^b^ D:Not specified
6	Chen	2021	PA (multi-component exercise)	Weight-bearing device	Exercise specialists	Group class	Physical fitness (baseline level of fitness)	C:12 wks F:3 D:30 min
7	Jiayuan	2021	Mind–body intervention PA (multi-component exercise)	No materials needed	Exercise specialists	Group class	No	C:6 months F:2 D:60 min
8	Mrurkesu	2021	PA (multi-component exercise^c^) Nutrition education Cognitive training Psychosocial support	Ball (for exercise) Cognitive challenge worksheet	Exercise specialist	Group class	No	C:12 wks F:2 D:Not specified
	Kwan	2021	PA (cycling) Cognitive training VR	Technological device Cycle	Non specialist	Individual training	Physical performance in previous session	C:8 wks F:2 D:30 min

^a^ 2 groups of chair-based exercises: a chair elastic band muscle strength exercise and a chair multimodal exercise

^b^ Sessions/week depend on the baseline fitness, each session is present as a 10-min brisk walking session

^c^ A multi-component exercise that includes progressive resistance training, aerobic, balance, and flexibility training

^d^ Programme included strength, balance, and flexibility training activities

^e^ Borg Rating of Perceived Exertion

### Components of intervention

Seven types of interventional components were found in the included studies. They were: physical activity, cognitive training, nutrition education, behavioural interventions, mind–body interventions, psychosocial support, and virtual reality (VR).

#### Physical activity

All nine studies included a physical activity component in the intervention (*n* = 9, 100%). Four studies (44.4%) [22, 23, 39, 40] used a multi-component exercise intervention, which included strength, balance, and flexibility training [22, 23], Otago exercise [39], and Tai Chi Chuan [40]. Two studies (22.2%) [24, 25] used a strengthening exercise: one was chair-based [25], and the other was resistance-based training [24]. One study (11.1%) focused on brisk walking [20] with one study focused on cycling (11.1%) [41]. High-speed power exercise training was used in one study (11.1%) [21].

#### Cognitive training

Two studies (*n* = 2, 22.2%) [23, 41] included cognitive training components in the intervention. The aims of the interventions in those studies were to enhance short-term memory, attention, information-processing skills, perceptual organizational tasks, reasoning and logic, and problem-solving abilities through the use of “Pen to Paper” tasks such as jigsaw puzzles and matrix reasoning [23], and through video games of daily living tasks such as finding a bus stop and reporting lost items [41].

#### Nutrition education

The nutritional component was included in one study (*n* = 1, 11.1%) [23]. In that study, the nutritional intervention was run during dietary counselling with an educational approach. It aimed to reduce the risk of malnutrition in older people by encouraging healthy eating habits.

#### Behavioural interventions

One study (*n* = 1, 11.1%) [20] included an mhealth behavioural intervention for the intervention group. The behavioural intervention consisted of motivational interviewing and regular telephone support through the self-tracking of walking behaviours, e-reminders, and real-time feedback.

#### Mind–body interventions

One study (*n* = 1, 11.1%) [40] carried out Tai Chi Chuan with mindfulness training.

#### Psychosocial support

One study (11.1%) [23] used group-based intervention to promote social participation. The aim was to enhance the self-esteem, self-achievement, self-worth, and self-efficacy of older people.

#### Virtual reality

One study (11.1%) [41] used a VR platform to carry out motor-cognitive training. This provided a virtual environment of daily living to simulate real-life scenarios in a controlled, safe setting for training.

#### Materials used

Elastic bands (*n* = 3, 33.3%) [21, 24, 25], chairs (*n* = 2, 22.2%) [22, 25], weight-bearing devices (*n* = 2, 22.2%) [22, 39], technology devices (*n* = 2, 22.2%) [20, 41], balls (*n* = 1, 11.1%) [23] and ergometers (*n* = 1, 11.1%) [41] were used in carrying out physical training interventions. Two studies used technological devices to carry out the intervention, for example, one study (11.1%) used smartphone technology, to adopt the mHealth function, as a monitoring and communication device as the intervention to evaluate the changing physical activity behaviour [20]. One study (11.1%) used an immersive virtual reality system and an ergometer to simulate daily living activities, such as grocery shopping and countryside travelling [41].

#### Providers

Two types of intervention providers were used in the included studies. Six studies (66.7%) [21, 23–25, 39, 40] used exercise specialists, such as a physiotherapist. Two studies featured non-specialist interventionists (22.2%) [20, 41], and one study failed to provide details of the interventionist [22].

#### Mode of delivery

Two delivery formats were found: group (*n* = 4, 44.4%) or individual training (*n* = 4, 44.4%). One study used an mhealth system for online coaching.

#### Tailoring

Tailoring the intensity of the training according to the physical fitness of the individual was the most widely adopted method (*n* = 7, 77.8%). Different strategies for assessing physical fitness were used in the identified studies: measured against baseline fitness, using 1) The Borg Rating of Perceived Exertion (RPE) (*n* = 3, 33.3%), 2) Maximum heart rate (HRmax) (*n* = 1, 11.1%), 3) a baseline level of fitness (*n* = 2, 22.2%), and 4) performance in a previous session (*n* = 1, 11.1%).

#### Dosage

In the included studies, the intervention lasted from 8 weeks to 24 months, with around 2–6 sessions weekly. Each session lasted around 30–60 min.

### Objective two: the effects of different types of interventions on cognitive functions, frailty, and physical functions in older people with cognitive frailty

#### Effect on cognitive function

Five studies [20, 21, 24, 40, 41] demonstrated positive outcomes on cognition, with effect sizes ranging from 0.345 to 1.19. However, in the subgroup analysis of cognition in Lee, et al.’s study [21], mental flexibility, self-control of behaviour, inhibitory control, and primitive reflex were not significant, with effect sizes ranging from 0.11 to 0.14.

**Fig. 2:**
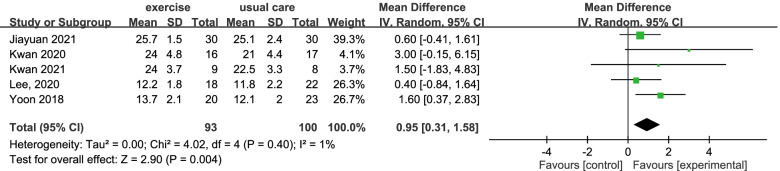
The effect on cognitive function after different types of interventions

The between-group effect on the cognitive function of the intervention group was analysed by a meta-analysis of five studies involving a total of 193 subjects (Fig. 2) and as a result, low heterogeneity was found among the included studies (I^2^ = 1%). The overall between-group mean difference was 0.95. The 95% CIs ranged from 0.31 to 1.58, showing that the interventions could significantly improve the cognitive function of the participants, compared with the findings for the control groups.

#### Effect on frailty status

**Fig. 3:**
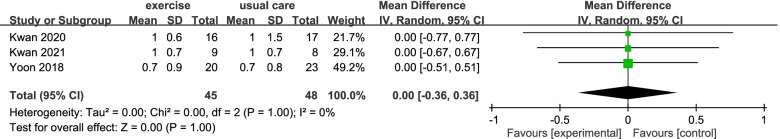
The effect on frailty scores after different types of interventions

Three studies [20, 24, 41] evaluated the effect on frailty status. All showed positive outcomes on the Fried frailty index with effect sizes ranging from 0.97 to 1.48. The between-group effect on the frailty status of the intervention group in three studies was analysed, which included 93 subjects with a similar low heterogeneity among the included studies (I^2^ = 0%) (Fig. 3). The overall between-group mean difference was zero, and the 95% CI ranged from -0.36 to 0.36. Although the effect size was positive, the meta-analysis showed no significant statistical improvement in frailty status after the interventions.

#### Effect on physical function

Eight studies [20, 21, 23–25, 39–41] showed a positive effect on physical functions such as gait speed, TUG, and hand grip strength. The effect size on the following physical functions was: gait speed 0.283—4.11, TUG 0.62—2.22, handgrip strength 0.20—1.76, gait speed 1.46, peak torque 0.19—0.4, and rate of torque 0.32—2.47. The daily activity function was reflected by FAQ and PASE. The effect size of FAQ was 3.62, and that of PASE was 1.01.

**Fig. 4:**
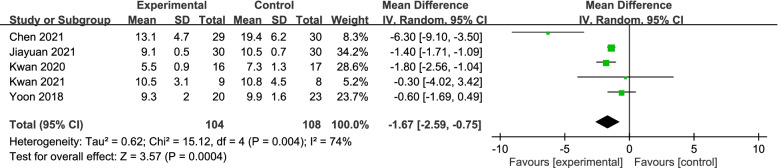
The effect on physical functions (walking by TUG) after different types of interventions

The between-group effect of different interventions on walking by TUG in four studies was analysed involving a total of 212 subjects. As shown in Fig. 4, the overall between-group mean difference was 1.67, and the 95% CI ranged from -0.75 to -2.59, showing that the interventions significantly improved the physical functions of the participants compared with the findings for the control groups. High heterogeneity (I^2^ = 74%) was found; therefore, no reliable result on this outcome can be generated.

**Fig. 5:**
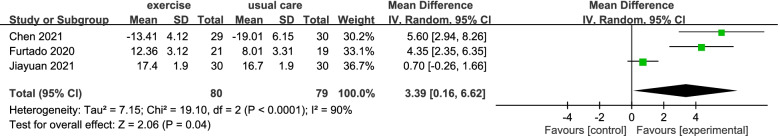
The effect on physical functions (Core strength by Chair and Stand Test) after different types of interventions

In Fig. 5, the between-group effect on the physical function of leg strength and endurance was assessed by the Chair and Stand Test in three studies, that included 159 subjects. The overall between-group mean difference was 3.39. The 95% CI ranged from 0.16 to 6.62, showing that the interventions improved performance in the Chair and Stand Test compared with the findings for the control groups. However, high heterogeneity was found among the included studies (I = 90%); thus, no reliable result on this outcome can be generated.

#### Effect on physical activity

In the intervention group, the effect on physical activity was statistically significant about time spent on walking, step count, and MVPA in Kwan’s study (2020) [20] and IPAQ in Murukesu’s study (2021) [23], with effect sizes ranging from 0.28 to 0.59. Details of the within-group effect size (Hedges G) for the intervention group at T1 are shown in Table 4.

**Table 4:** Results of the individual studies

No	Author	Year	Outcome	Measurement	Effect size—within group (Hedges G)
					T1
1	Liu	2018	Immune marker	IL6	NA
			Cognitive frailty status		NA
2	Yoon	2018	Cognition	MMSE	NA
			Cognition	FAB	0.73
			Cognition	CERAD	NA
			Cognition	TMT-A	0.21
			Cognition	TMT-B	0.44
			Cognition	DS	0.34
			Cognition	RM	0.73
			Frailty Status	FFI	0.97
			Physical function	TUG	0.62
			Physical function	SPPB	0.79
			Physical function	Inhibitory control	4.11
			Physical function	Hand grip strength	1.76
			Physical function	PT	0.19
			Physical function	RTD	0.32
3	Furtado^a^	2020	Immune marker	IL6	0.18
			Physical function-SFTB	30 s chair and stand test	1.02
			Physical function-SFTB	30 s arm curl test	1.06
			Physical function-SFTB	2 min step test	0.69
			Physical function-SFTB	chair seat and reach	0.39
			Nutrition	MNA	NA
4	Kwan	2020	Cognition	MoCA	0.47
			Frailty status	FFI	1.48
			Physical activity	WT (min/Day)	0.54
			Physical activity	SC (Step/Day)	0.59
			Physical activity	MVPA	0.319
			Physical activity	PASE	1.01
			Physical function	Hand grip strength	0.52
			Physical function	Gait speed	1.46
5	Lee	2020	Cognition	MMSE	NA
			Cognition	FAB	0.345
			Cognition-FLT	Conceptualization	0.26
			Cognition-FLT	Mental flexibility	0.14
			Cognition-FLT	Motor programming	0.76
			Cognition-FLT	Self-control of behaviour	0.13
			Cognition-FLT	Inhibitory control	0.11
			Cognition-FLT	Primitive reflex	0.14
			Frailty status	FFI	NA
			Physical function	TUG	NA
			Physical function	Gait speed	NA
			Physical function	PT	0.40
			Physical function	RTD	2.47
6	Chen	2021	Physical function	FTSST	0.62
			Physical function	TUG	0.64
			Physical function	Berg balance score	3.78
			Psychosocial fitness	GDS-15	0.31
			Psychosocial fitness	SF-12 MCS	0.30
7	Jiayuan^b^	2021	Cognition	MMSE	0.76
			Physical function	SPPB	0.69
			Physical function	TUG	2.22
			Physical function	30 s chair test	0.66
8	Murukesu	2021	Physical activity	IPAQ	0.28
			physical function	FAQ	3.62
			Psychosocial fitness	Flourishing scale	0.17
				GHQ-12	0.06
				COPE	0.16
9	Kwan	2021	Cognition	MoCA	1.19
			Frailty status	FFI	1.29
			Physical function	TUG	1.07
			Physical function	Hand grip strength	0.20

*CERAD* Consortium to Establish a Registry of Alzheimer’s Disease, *COPE* Coping Orientation to Problems Experienced, *DS* Digit span test, *FAB* Frontal Assessment Battery, *FAQ* Functional Activity Questionnaire, *FLT* Frontal lobe test, *FFI* Fried Frailty Index, *FTSST* Five time sit to stand test, *GDS-15* Geriatric Depression Scale, *GHQ-12* General health questionnaire, *IPAQ* International physical activity questionnaire, *MMSE* Mini Mental State Examination, *MNA* Mini Nutritional Assessment, *MoCA* Montreal Cognitive Assessment, *MVPA* Moderate to vigorous physical activity, *PASE* Physical Activity Scale for the Elderly, *PT* Peak torque, *RM* Rey 15-item memory test, *RTD* Rate of torque, *TMT* Trail Making Test, *TUG* Time Up and Go Test, *SC* Step count, *SF-12 MCS* 12-item Short Form Health Survey Mental Component Summary, *SFTB* Senior Fitness Test Battery, *SPPB* Short Physical Performance Battery, *WS* Walking time

^a^ CME used as the main intervention group

^b^ mindfulness intervention used as the control group and MTCC as the main intervention group

## Discussion

This is the first systematic review to report the effect of interventions on older people with cognitive frailty. There are three key findings in this review: 1) all studies employed physical activity as one of the intervention components and many of the physical activity components were implemented along with other components, 2) the interventions were effective at improving global cognitive function and physical functions, and 3) the interventions were not effective at treating physical frailty.

All interventions included physical activity components, for example, walking, high-speed power training, and flexibility training along with additional components such as behavioural interventions and nutritional education). Ageing resulted in a decrease in exercise capacity, muscle strength, flexibility, and bone mass. These changes led to a decrease in physical functioning, a decrease in the ability to carry out activities of daily living, and poorer quality of life [42]. Interventions focusing on physical activity can enhance physical functioning and cardiorespiratory fitness [42, 43]. Previous studies suggested that physical activity could modify neurobiological conditions associated with cognitive frailty, such as insulin resistance, cerebral glucose metabolism, and sarcopenia [44, 45]. Physical inactivity is a modifiable risk factor for cognitive frailty [10, 18].

The meta-analysis demonstrated that interventions with physical activity as one of the components can significantly promote global cognitive and physical functions compared with controls. Similar findings were also observed in other reviews on dance interventions for older people [46] and high-intensity and frequent resistance exercises for those people with mild cognitive impairment [47]. Physical activity interventions have positive effects on brain structure, function, and connectivity by neurogenesis and angiogenesis [43, 48]. For example, an increase in cardiorespiratory fitness has resulted in slowing the rate of grey matter loss [43, 49]. One systematic review showed that physical training is effective in increasing muscle strength and muscle mass in older people with physical frailty [50]. The findings of our review are consistent with previous reviews inasmuch that physical activity is effective at improving or delaying cognitive decline in older people, as well as at promoting walking ability [51–53]. This review also suggests that interventions with a physical activity component are effective in promoting global cognitive function and physical function in older people with cognitive frailty. However, the efficacy of an individual component (e.g., physical activity, nutrition education, behavioural interventions) on cognitive and physical function could not be concluded in this review. This is because different studies employed different combinations of components, and these components were controlled sporadically. Future studies should examine the efficacy of different components on cognitive and physical function collectively. As a result, a more precise recommendation could be provided in the development of clinical guidelines to treat older people with cognitive frailty. In addition, the potential synergistic effects of other components (e.g., behavioural interventions, nutritional education) added to physical activity should also be examined.

Surprisingly, our meta-analysis does not support the argument that an intervention with a physical activity component could lead to a reduction in frailty. This contradicts previous reviews that suggest interventions using physical activity could ameliorate physical frailty [54–56]. The possible reasons for this discrepancy are threefold. First, even with the meta-analysis, the total number of participants in this study was small. The potential effect of the interventions on physical frailty could not be detected with the given sample size. Second, a dose–response relationship is known to exist between physical functions and the amount of physical activity [57]. The duration of the interventions of the studies included in the meta-analysis was from 8 to 16 weeks. Previous systematic reviews suggested that a minimum duration of 10 weeks is needed to yield positive frailty outcomes among frail older people [58] and at least 12 weeks among prefrail older adults [28]. The length of the intervention might be too short or the intensity is not strenuous enough to yield statistically significant effects to achieve improvements in physical frailty. In the literature, aerobic, resistance and flexibility training were recommended for use in treating frailty, but the efficacy of the different types of physical activity interventions on physical frailty varied and their effects on physical frailty are not well known [59]. In the meta-analysis, the types of physical activity and the number of intervention components differed. This heterogeneity in physical activity and doses employed might have led to the inconclusive effect. Last, it is known that the level of frailty at baseline has an impact on the effectiveness of a physical activity intervention [60]. All three studies in the meta-analysis included both pre-frail and frail older people. The heterogeneous baselines in frailty level may have led to an inconclusive effect. Also, this result may indicate that the effect of the interventions may only be useful to a specific group of persons. The generalisability of the interventions to people with different levels of frailty at baseline is in doubt. More studies are needed to identify the appropriate types and doses of interventions to treat older people with cognitive frailty, as well as their effects on those with different levels of severity of cognitive frailty at baseline. Additionally, further studies are required to investigate the difference in effect on physical frailty in the frail and cognitively frail population.

This systematic review has important implications for future research and practice. In general, most of the included studies were of good quality, yet blinding of the assessors was not done, and the formal sample size estimation based on power analysis was not conducted in some studies. One study did not report adequate information for meta-analysis. Future studies should address the limitations of those identified in individual studies to strive for better reporting of methods and findings. Cognitive frailty is found to be a significant predictor of all-cause mortality and dementia [28]. The findings of this review brought to light the potential future development of effective interventions to combat the growing problem of cognitive frailty. Also, there is too little information about the effects of other intervention ingredients (such as nutrition, psycho-social, and medical interventions) and these ingredients need to be addressed by future studies.

There were several limitations in this review. First, the heterogeneity of the meta-analysis of physical functions was high. This may be due to the intervention components used in the different studies and the instruments measuring the same outcome varied, although we attempted to minimize the heterogeneity by setting clear inclusion and exclusion criteria. Second, some of the studies employed only an active control without using the usual care. The meta-analysis might have underestimated the effect of the interventions.

## Conclusion

This review showed that some interventions had a positive effect on cognitive function and physical function in terms of walking and core strength, but had no effect on physical frailty inasmuch that physical activity is the essential component of the intervention. It is recommended that physical activity be a compulsory component of these types of interventions for older people with cognitive frailty. Further studies should be conducted to examine the optimal type, dosage, and setting of the physical activity intervention and to further explore the effectiveness of such interventions on the frailty status of older people with cognitive frailty.

## Supplementary Information

**Additional file 1: Appendix 1.** Search strategy in databases.

## Data Availability

The datasets during and/or analysed during the current study are available from the corresponding author on reasonable request.

## Abbreviations

VR: Virtual reality
MMSE: Mini-Mental State Examination
FAB: Frontal Assessment Battery
TMT: Trail Making Test
MoCA: Montreal Cognitive Assessment
FFI: Fried Frailty Index
