# Supplementary material for: The effects of interventions to enhance cognitive and physical functions in older people with cognitive frailty: a systematic review and meta-analysis
Source: Eur Rev Aging Phys Act. 2022 Aug 24;19:19. doi: 10.1186/s11556-022-00299-9 (PMC9400290; doi:10.1186/s11556-022-00299-9)
Supplement: Supplementary file 1 — Additional file 1: Appendix 1. Search strategy in databases. [file 11556_2022_299_MOESM1_ESM.docx]

# Appendix 1. Search strategy in databases

| Source | Terms searched | Results  (articles) |
| --- | --- | --- |
| Pubmed | **"Cognitive frail*” [All Fields] OR (("cognitive impairment*"[All Fields] OR "mild cognitive impairment*"[All Fields]) AND ("frail*"[All Fields] OR frailty" [Mesh] OR "pre-frail*"[All Fields] OR "frailty syndrome"[All Fields] OR "frail elderly" [Mesh] OR "frail elderly"[All Fields] OR "frail older adult*[All Fields]))**  **AND**  “elder*” **[All Fields]** OR “older adult*” **[All Fields]** OR “older elder*” **[All Fields]** OR “geriatric*” **[All Fields]** OR “geriatrics” [Mesh] OR “old age” **[All Fields]** OR “old people”**[All Fields]** OR “senior*” **[All Fields]** OR “ageing*” **[All Fields]** OR “aging” **[All Fields]** OR “aging” [Mesh] OR “aged” **[All Fields]** OR “aged” [Mesh]  AND  Limited to "randomized controlled trial" or “clinical trial” or “meta-analysis” or “systematic reviews” and “aged 45+ years” | 13 |
| EMBASE | **"Cognitive frail*" OR (("cognitive impairment*" OR "mild cognitive impairment*" OR “mild cognitive impairment/exp”) AND ("frail*" OR "pre-frail*" OR "frailty syndrome" OR “frail elderly/exp” OR "frail elderly" OR "frail older adult*”))**  **AND**  “elder*” OR “older adult*” OR “older elder*” OR “geriatric*” OR “geriatrics/exp” OR “old age” OR “old people” OR “senior*” OR “ageing*” OR “aging*” **OR “aging/exp” OR aged OR “aged/exp”**  AND  Limited to “clinical study” or “Clinical trial” or “clinical trial topic” or “comparative study” or “controlled clinical trial” or “controlled study” or “evidence based practice” or “feasibility study” or “intervention study” or “major clinical study” or “meta analysis topic” or “multicenter study” or “pilot study” or “prospective study” or “randomized controlled trial” or randomized controlled trial topic” or “systematic review” | 611 |
| PsycINFO | **"Cognitive frail*" OR (("cognitive impairment*"** OR “cognitive impairment/exp” **OR "mild cognitive impairment*" OR “mild cognitive impairment/exp”) AND ("frail*" OR “frail/exp” OR "pre-frail*" OR "frailty syndrome" OR "frail elderly" OR "frail older adult*”))**  **AND**  “elder*” OR “older adult*” OR “older elder*” OR “geriatric*” OR “old age” OR “old people” OR “senior*” OR “ageing*” OR “aging*” **OR “aging/exp”** OR “aged” **OR “aged/exp” O**R 'citizen*' OR 'citizen/exp' OR 'pensioner*' OR 'pensioner/exp'  AND  Limited to “empirical study” or “quantitative study” or “prospective study” or “systematic review” or treatment outcome” or “clinical trial” or “meta analysis” | 157 |
| CINAHL | **(TX "Cognitive frailty" OR (("cognitive impairment*"** **OR "mild cognitive impairment*" OR “mild cognitive impairment/exp”) AND ("frail*" OR "pre-frail*" OR "frailty syndrome" OR “frailty syndrome/exp” OR "frail elderly" OR “frail elderly/exp” OR "frail older adult*”)))**  AND  (TX “elder*” OR “older adult*” OR “older elder*” OR “geriatric*” OR “geriatrics/exp” OR “old age” OR “old people” OR “senior*” OR “ageing*” OR “aging*” OR **“aging/exp”** OR “aged” OR “aged/exp”)  AND  Limited to “middle aged 45+ years” | 320 |
| The Cochrane Library  (CENTRAL) | **"cognitive frailty" OR (("cognitive impairment*"** **OR "mild cognitive impairment*") AND ("frail*" OR "pre-frail*" OR "frailty syndrome" OR "frail elderly" OR "frail older adult*”))**  **AND**  “elder*” OR “older adult*” OR “older elder*” OR “geriatric*” OR “old age” OR “old people” OR “senior*” OR “ageing*” OR “aging*” OR “aged” | 133 |
| Medline | **"cognitive frailty" OR (("cognitive impairment*"** **OR "mild cognitive impairment*") AND ("frail*" OR "pre-frail*" OR “frailty [Mesh] OR "frailty syndrome OR "frail elderly" OR “frail elderly [Mesh]” OR "frail older adult*”))**  AND  “elder*” OR “older adult*” OR “older elder*” OR “geriatric*” OR “geriatrics [Mesh]” OR “old age” OR “old people” OR “senior*” OR “ageing*” OR “aging*” OR **“aging [Mesh]”** OR “aged” OR “aged [Mesh]”  **AND**  **Limited to “aged 45+ years”** | 944 |
